# Efficacy of Traditional Chinese Medicine Combined Online Group Psychotherapy (TCM-eRhab) on improving quality of life and relieving psychological burden for colorectal cancer survivors: a study protocol for a phase-II randomized controlled trial

**DOI:** 10.1186/s12906-024-04533-y

**Published:** 2024-07-30

**Authors:** Yunzi Yan, Jiaxi Liu, Ying Pang, Zixu Wang, Rongyan Peng, Demei Jiang, Yufei Yang, Lili Tang, Lingyun Sun

**Affiliations:** 1 Xiyuan Hospital, China Academy of Chinese Medical Sciences, Beijing, China; 2 Beijing University of Chinese Medicine, Beijing, China; 3 Beijing Cancer Hospital, Beijing, China; 4 Institute of Basic Research in Clinical Medicine, China Academy of Chinese Medical Sciences, Beijing, China

**Keywords:** Colorectal cancer, Psychological burden, Quality of life, Traditional Chinese medicine, Randomized controlled trial

## Abstract

**Background:**

More than 50% of colorectal cancer(CRC) patients experience cancer-related psychological burden after radical surgery, which can seriously affect their physical wellness, quality of life and even survival outcomes. Our research team developed a six-week Traditional Chinese Medicine Combined Online Group Psychotherapy (TCM-eRhab) and proved its efficacy on relieving cancer-related anxiety, depression and fear of cancer recurrence though phase I single arm clinical trial (*n* = 40). Large sample size randomized controlled clinical trial(RCT) is necessary to further evaluate TCM-eRhab’s role on improving quality of life and survival outcomes among this population.

**Methods:**

We design a phase II RCT study, in which 210 CRC patients who have received radical surgery (stage I-III) will be recruited. Eligible patients will be randomly assigned to TCM-eRhab group or usual care group by 2:1 ratio. Patients in the intervention group will receive the structured TCM-eRhab program for six weeks, while patients in control group will receive usual care only. The primary outcomes are quality of life, severity of anxiety, depression and fear of cancer recurrence. Cancer recurrence rate will also be calculated according to long term follow-up data.

**Discussion:**

As one of the first RCTs to evaluate the impacts of TCM combined psychological therapy to improve CRC patients’ quality of life after surgery, the results from this study will provide innovative knowledge and evidence on integrating TCM into CRC survivorship care and mind–body intervention model.

## Introduction

Colorectal cancer(CRC) accounts for more than 55,000 new cancer survivors and more than 29,000 deaths per year in China [1]. The 5- year survival rate is 60%-80% for early-stage colorectal cancer. Thus, attenuating postoperative recurrence risk is the best possible chance of cure for CRC patients. For early-stage CRC patients, surgical resection and adjuvant therapy is the curial curative intervention. However, cancer survivors are left with long-term physical and psychosocial morbidities because of cancer treatments and worries on cancer recurrence, which would eventually impair their quality of life [2]. Anxiety is one of the most common psychological symptoms, which’s prevalence rate among CRC patients was up to 29.0%, especially in patients with ostomy. In addition, the change of cognitive behavior leads to patients’ incorrect or distorted understanding, negative views and attitudes towards themselves [3]. Meanwhile, many studies have shown a strong correlation between cognitive intervention disturbance and anxiety [4, 5]. Consequently, psychosocial supports and symptom management for CRC survivors after radical surgery are essential to help them return to normal life.

According to NCCN guidelines, screening for cancer survivors’ psychological burden and providing education, group session and psychotherapy are recommended [6]. Existing studies have showed that cognitive behavior therapy, group courses and reminiscence therapy were effective to reduce CRC survivors’ fear of cancer recurrence(FCR) and psychological symptoms [7]. Growing evidence is also demonstrating that certain integrative oncology interventions, such as yoga, meditation and acupuncture could be valuable to wholistically promote CRC survivors’ rehabilitation on both body and mind [8, 9]. However, the level of evidence is still insufficient to apply these interventions into clinical practice guidelines and standard of cancer care.

Traditional Chinese Medicine(TCM) is an important component of integrative medicine, which could provide valuable guidance on lifestyle and coping skills for CRC survivors after surgery. Previously, our research team had developed an online program in which TCM theories and methods were integrated into conventional group psychotherapy(TCM-eRhab) [10]. Through pilot phase I clinical study, we preliminarily proved that the six-week TCM-eRhab program was feasible for clinical utilization and could significantly reduce CRC survivors’ FCR and psychological symptoms including anxiety, depression and insomnia. We also found that such intervention also could modulate certain gut microbiome that associated with psychological disorders. However, statically significant changes on quality of life had not been observed after intervention due to limitation on small sample size of our pilot study (*n* = 40). Thus, although TCM-eRhab has a significant potential as an effective intervention for CRC survivors, further evaluation in larger RCT trials on TCM-eRhab’s efficacy for their postoperative quality of life, anxiety and depression is required.

The primary aim of this current RCT study is to evaluate TCM-eRhab’s role on improving quality of life among CRC survivors while relieving psychological burdens when compared with usual care control group. We also plan to assess its association with patients’ survival outcomes especially disease-free survival and discover the potential beneficial population of such intervention.

## Material and methods

### Study design and setting

**Fig. 1:**
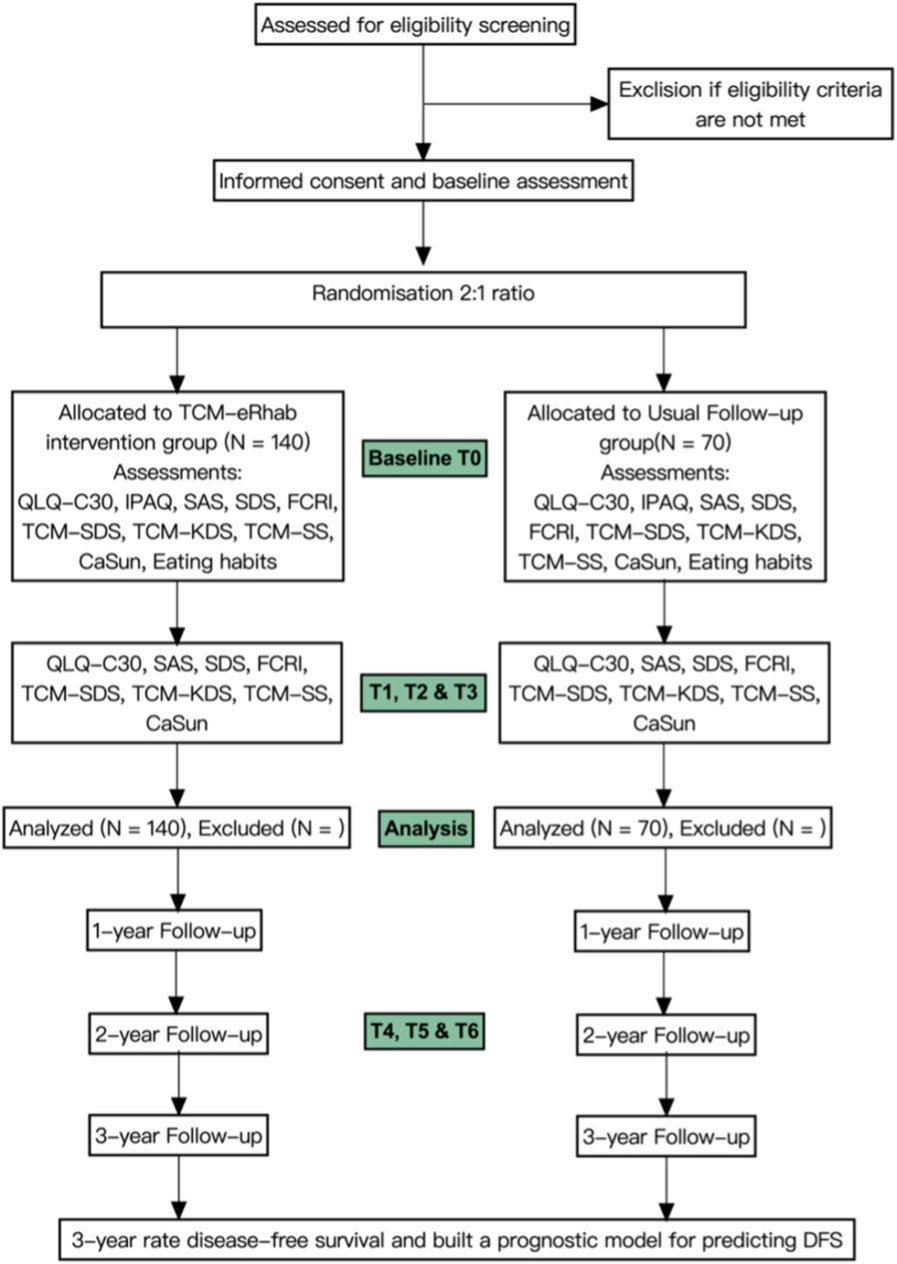
Participant Flow

This is a prospective, open label, parallel group randomized clinical trial with interventions designed to assess quality of life, anxiety, and depression outcomes according to Traditional Chinese Medicine Combined Online Group Psychotherapy(TCM-eRhab) program among early-stage CRC patients. We plan to enroll 210 patients in Xiyuan Hospital, China Academy of Chinese Medical Sciences(CACMS) and Beijing Cancer Hospital. During the screening visit, study procedures are explained and written informed consent is obtained by study researcher. Figure 1 depicts the flow of patients through the study. Trial registration is available at International Traditional Medicine Clinical Trial Registry (ITMCTR2022000041). The protocol has been approved by Ethics Committee of Xiyuan Hospital, CACMS(2022XLA079-1).

### Inclusion criteria/Exclusion criteria

The trial will be performed with CRC patients who meet the following eligibility criteria: between 18 and 75 years of age; CRC patients who have undergone radically surgery and histologically confirmed AJCC TNM stage I, II and III disease. To ensure the communication is fluency for CRC patients, patients should be signed written informed consent.

As the study has a significant focus on the stage I-III CRC patients, if the patients have had any evidence of metastatic disease, then they will be excluded from the study. Patients who have history of clinically relevant psychiatric disability (e.g., severity personality disorders), precluding informed consent. If either person is unable to have a good compliance, they will be excluded from the study.

### Randomization and allocation

Following baseline assessment and registration, patients will be randomized in a 2: 1 ratio to either the TCM-eRhab program or to the normal usual care control group, using a computer-generated randomization list. Randomization will be overseen by research assistant, who will have no direct involvement in implementing the trial. Randomization is stratified by clinical center.

### Interventions

#### TCM-eRhab intervention group

The treatment will be jointly conducted by the department of psychology rehabilitation at Beijing Cancer Hospital, TCM-psychology department at Clinical Basic Research Institute of CACMS and TCM-oncology department at Xiyuan hospital. The feasibility of this protocol has been established by our previous trial, which will last for 6 consecutive weeks, once a week, lasting 60 min to 90 min each. The intervention protocol will be divided into six subjects, namely (1): Nice to meet you. (2): How to manage our symptoms? (3): How to manage our emotions? (4): How to be friend with our body? (5): How to support out family and loved ones? (6): How to plan out future and farewell! The content of TCM-eRhab in each week is detailed in Table 1. Then, the physical symptoms of patients will be personalized treatment by the TCM-oncologist and psychology therapist. The physical symptoms management of TCM and western medicine intervention in Table 2.

**Table 1:** Session structure of TCM-eRhab program

**Period**		**Subjects**	**Intervention instructions**	**Technics**
**Week 1**	**Initial Phase**	*Session1:* Nice to meet you	Self-introduction; What is the aim rehabilitation in TCM concept (harmony); Mindfulness practice; guide patients to care themselves and share their feelings	Expression support
**Week 2**		*Session2:* How to manage our symptoms?	How to modify lifestyle after CRC diagnosis? How to use TCM to preserve life? Why we experience symptoms and how could we use TCM to manage them?	Cognitive Training;
**Week 3**	**Work Phase**	*Session3:* How to manage our emotions?	Do you fear of cancer recurrence? How could we manage emotion and stresses? Concentration practice. Acupoints and acupressure	Expression support
**Week 4**		*Session4:* How to be friend with our body?	Can you communicate with your body? Organ scan and mindfulness practice	Cognitive Training
**Week 5**	**Final Phase**	*Session5:* How to support out family and loved ones?	Anything changed on your family and social support after cancer diagnosis? Five most important things in your life. Relax practice	Cognitive Training
**Week 6**		*Session6:* How to plan out future and farewell!	Love mindfulness practice; future plan; lucky diary; farewell	Expression support

**Table 2:** The physical symptoms management of TCM and western medicine intervention

Syndromes	Instrument	TCM-intervention	Western Medicine-intervention
Defecation obstacles	FACT-C	(1) The TCM syndrome differentiations. (2) Acupuncture (3) Acupoint pressure therapy	(1) Intestinal flora regulator. (2) Prokinetic agents
CIPN	CIPN-20	(1) The TCM syndrome differentiations. (2) Acupuncture (3) Chinese herb bath	(1) Neurotrophic drugs. (2) Psychoactive drugs
Sleep disturbance	PSQI	(1) The TCM syndrome differentiations. (2) Acupuncture (3) The TCM Five-element Music Therapy	(1) Cognitive training. (2) Psychoactive drugs. (3) Meditation and relax training
Cancer related fatigue	BFI	(1) The TCM syndrome differentiations. (2) Tai Chi and Qigong	(1) Relax training. (2) Exercise training
Anxiety and Depression	HADS	(1) The TCM syndrome differentiations. (2) Acupuncture (3) The TCM Five-element Music Therapy	(1) Psychological consultation. (2) Anti-anxiety Anti-depressant. (3) Meditation and relax training

#### Usual care (Control) group

The control group will not be invited to participate in the TCM-eRhab intervention group at the first six weeks. However, they will be compensated with TCM-eRhab treatments afterwards according to their own willingness. According to NCCN guideline, patients will do laboratory chemistries plus complete blood count (CBC), tumor markers, abdominal US (ultrasound) and CT scan during the first 3 years.

### Primary outcomes

#### Quality of life

Quality of life will be measured subjectively using the Quality of Life Questionnaire-Core (QLQ-C30), which as a core questionnaire has been used in a wide range of cancer clinical trials [11]. It is supplemented by disease specific modules. Categories include functional scales, global health status and QOL scale, in addition to several single-item symptom measures [12, 13]. In the current study, QLQ-C30 will be collected via online app with patients at baseline, week 2, 4 and 6 during intervention or usual care phase (Table 3).

**Table 3:** Study assessment procedures and timetable

Visit schedule	pre	Treatment phase			Follow-up phase		
Study phase	Baseline testing	T1	T2	T3	T4	T5	T6
Week/year	Baseline	Week 2	Week 4	Week 6	1 year	2 years	3 years
**General data**							
Eligibility screening	√						
Informed consent	√						
Baseline information	√						
IPAQ scale	√						
**Efficacy parameters**							
SAS	√	√	√	√			
SDS	√	√	√	√			
FCRI	√	√	√	√			
QLQ-C30	√	√	√	√			
TCM-SS	√	√	√	√			
TCM-SDS/TCM-KDS	√	√	√	√			
CaSUN	√	√	√	√	√	√	√
Caner recurrence/other outcome events					√	√	√
**Safety parameters**							
Adverse events		√	√	√			
End of treatment				√			

#### Self-reported psychological stress

##### The Self-Rating Anxiety Scale (SAS)

The scale includes 20 items and have four grades (Grade I-IV), ranging from I, which considers anxiety from normal, to Grade IV, which describes the most severe anxiety of the patients [14].

##### The Self-Rating Depression Scale (SDS)

To assess the severity of depressive symptoms, the self-rating depression scale is used. The scale consists of 20 items [15, 16], measuring the severity of depressive symptoms on a four-point Likert scale.

##### Fear of Cancer Recurrence Inventory (FCRI)

We use the fear of cancer recurrence inventory (FCRI) to evaluate the participants’ fear of recurrence (FCR). FCRI is a 42-item scale which is recognized as one of the psychometrically strongest measures of FCR [17–19]. Higher scores indicate a greater level of psychological distress.

### Secondary outcomes

As secondary outcomes, The Cancer Unmet Needs (CaSun), Traditional Chinese Medicine syndrome scale (TCM-SS) and TCM spleen deficiency scale (TCM-SDS) will be applied. Another secondary efficacy endpoint of the study is Disease Free Survival (DFS), defined as the time from the date of randomization up to the date of first local, regional, or distant relapse. The follow-up will begin when the patient completes treatment phase. The follow-up visits will be done every year lasting for 3 years. Patients will do abdominal US (ultrasound) and CT (Computerized Tomography) and conduct the information including lifestyle, psychological state, and social relations.

#### Safety assessment

All safety parameters will be recorded in terms of listings and summary tables. We don’t expect increased risks for patients participating in the treatments given the results of earlier studies. Thus, no specific hypotheses were formulated to incorporate in the analysis.

#### Sample size

Previous studies evaluating TCM-eRhab program for early-stage CRC patients reported a mean difference of 66.1 ± 10 points after the intervention on the QOL-C30 scale. In addition, the evidence reported on the average of scores of QOL-C30 scale for CRC patients with early-stage was 61.5 ± 10 points. In order to provide 80% power to detect this clinically meaningful difference, using a two-sided test with alpha = 0.05 and assuming that 15% of patients opt out, we need to approach approximately 210 patients (TCM-eRhab intervention group:140 patients, Control group: 70 patients).

#### Data management and smartphone app

The majority of the data is collected online using app and can only be accessed by the research team. The data management process will be complied with the regulatory requirements of Clinical Trial Quality Management Regulations and Clinical Trial Data Management Work Technical Guidelines to ensure the authenticity, integrality, accuracy, and traceability of data.

We have developed an app through WeChat Platform which customized for CRC patients with psychological rehabilitation needs(software copyright registration number: 2022SR1412831). The TCM-eRhab app will be interactive and incorporate individualized professional psychology rehabilitation course, lifestyle coaching support and TCM physical activity guidance. Through the TCM-eRhab app, study data will be collection and stored on the app and access only by our team. Furthermore, TCM-eRhab program videos can also be pushed to individuals, making the user’s experience on the APP a personalized one.

### Statistical analysis

Analysis of the study will be performed using SPSS statistical software package, version SPSS26.0 (Chicago, IL, USA) by the “intention-to-treat” principle, therefor all randomized patients satisfying eligibility criteria will be included in the efficacy analysis. A comparison of demographic, social and clinical characteristics will be carried out for each arm. The primary analysis of the QLQ-C30 questionnaire and Self-reported psychological pressure scales is the domain-averaged difference in scores between TCM-eRhab intervention group and usual follow-up group after intervention. Difference in these variables between the intervention and control groups will examined using either chi-square tests or analysis of variance. To examine whether anxiety, depression and clinical syndromes are the prediction factors of metastasis after intervention, modelling will be performed in Stata.

## Discussion

TCM-eRhab program as an online mind–body group therapy intervention has the potential to address complex constellation of symptoms. Meanwhile, TCM-eRhab program integrates treatment elements of TCM psychotherapy and modern psycho-oncology. By carrying out this randomized controlled trial, it will facilitate the development of current clinical practice towards innovative models of integrative Chinese and western rehabilitation and optimize the modern mind–body intervention methods.

The main objective of this study is to assess the evidence of TCM-eRhab program for improving CRC patient’s quality of life after radical surgery as well as benefiting their survival outcomes especially DFS. According to current ASCO guideline, promoting health condition, treating cancer related symptoms and preventing cancer recurrence are all major components of survivorship care for CRC patients. However, there is still lack of an effective and efficient intervention that could meet more patients’ needs at the same time. Thus, our study results will have profound meaning for the development an integrative intervention model of cancer survivorship care for CRC survivors.

This study will conduct online group therapy for patients which contains potentially unique therapeutic advantages such as encouraging expression, peer-support, coping skills, mindfulness practice and emotional expression. In addition, each patient will receive a comprehensive psychological, symptom and survivorship care needs assessment that is carried out at the beginning of the therapy, so that individualized TCM and supportive care plans can be integrated into the related session. So far, several studies have proved that education on lifestyle and health behavior has positive impact on CRC patients’ quality of life [20, 21]. To the best of our knowledge, the current study is one of the first to integrate methods and opinions of TCM into such education program and group therapy.

Experimental studies found that the combination of social interactions and cognitive stimulation regulated serum inflammatory factors, intestinal mucosal inflammatory factors and hypothalamic ghrelin levels [22, 23]. Some TCM herbal medicine, for example, Xiaoyaosan was found to be effective on preventing cancer cell progression and ameliorating gut dysbiosis through CRC mice model with chronic stress [24]. Our previous phase I trial also demonstrated that TCM-eRab intervention could modulate CRC survivors’ gut microbiome which had association with certain mental disorders. In the current trial, more mechanism on gut-brain axis interaction and CRC’s survival outcome are expected to be carried out.

There are some limitations on the current study design and certain obstacles for the administration of the clinical trial. First, as a behavioral study, it is unrealistic to conduct blind method, but we will use patient-reported outcome measurement to avoid investigator evaluation bias. Second, willingness to participate in the current trial is one of the major concerns for patients’ enrollment. According to an existing study, more than 60% CRC patients were not willing to participate in psychological intervention due to no perceived needs, older age and longer time since diagnosis [25]. Thus, in our study, we need to pay more attention on the balance between effectiveness of enrollment and population selection bias. Third, patients’ compliance of six-week intervention is also an important challenge for our study. We will record patients’ participation for each session and encourage them to comply with the protocol.

In conclusion, in this phase II randomized controlled clinical trial, we will evaluate the efficacy of an innovative model of online group psychotherapy, TCM-eRab on CRC survivors’ quality of life and survival outcomes after surgery. The implementation of the trial will help more CRC patients cope with their symptom burden and acquire guidance on healthy lifestyle by utilizing concepts and methods of TCM.

## Data Availability

All data of the clinical trial will be available under request to corresponding author (Lingyun Sun, slyslysun@126.com) after the primary results being published.
